# Hyperoxia-induced lung structure–function relation, vessel rarefaction, and cardiac hypertrophy in an infant rat model

**DOI:** 10.1186/s12967-019-1843-1

**Published:** 2019-03-18

**Authors:** Francesco Greco, Susanne Wiegert, Philipp Baumann, Sven Wellmann, Giovanni Pellegrini, Vincenzo Cannizzaro

**Affiliations:** 1 Department of Intensive Care Medicine and Neonatology, University Children’s Hospital Zurich, Steinwiesstrasse 75, 8032 Zurich, Switzerland; 2 Children’s Research Centre, University Children’s Hospital Zurich, Steinwiesstrasse 75, 8032 Zurich, Switzerland; 3 Zurich Centre for Integrative Human Physiology, Zurich, Switzerland; 4 Department of Neonatology, University Children’s Hospital Basel, Spitalstrasse 33, 4056 Basel, Switzerland; 5 Laboratory for Animal Model Pathology, Institute of Veterinary Pathology, Vetsuisse Faculty University of Zurich, Winterthurerstrasse 268, 8057 Zurich, Switzerland; 6 Present Address: Drug Safety and Metabolism, IMED Biotech Unit, AstraZeneca, Gothenburg, Sweden

**Keywords:** Hyperoxia, Bronchopulmonary dysplasia, Animal model, Respiratory system mechanics, Forced oscillation technique, Hysteresivity eta (η), α-Smooth muscle actin (α-SMA), Vascular endothelial growth factor (VEGF), Digital pathology

## Abstract

**Background:**

Hyperoxia-induced bronchopulmonary dysplasia (BPD) models are essential for better understanding and impacting on long-term pulmonary, cardiovascular, and neurological sequelae of this chronic disease. Only few experimental studies have systematically compared structural alterations with lung function measurements.

**Methods:**

In three separate and consecutive series, Sprague–Dawley infant rats were exposed from day of life (DOL) 1 to 19 to either room air (0.21; controls) or to fractions of inspired oxygen (FiO_2_) of 0.6, 0.8, and 1.0. Our primary outcome parameters were histopathologic analyses of heart, lungs, and respiratory system mechanics, assessed via image analysis tools and the forced oscillation technique, respectively.

**Results:**

Exposure to FiO_2_ of 0.8 and 1.0 resulted in significantly lower body weights and elevated coefficients of lung tissue damping (G) and elastance (H) when compared with controls. Hysteresivity (η) was lower due to a more pronounced increase of H when compared with G. A positive structure–function relation was demonstrated between H and the lung parenchymal content of α-smooth muscle actin (α-SMA) under hyperoxic conditions. Moreover, histology and morphometric analyses revealed alveolar simplification, fewer pulmonary arterioles, increased α-SMA content in pulmonary vessels, and right heart hypertrophy following hyperoxia. Also, in comparison to controls, hyperoxia resulted in significantly lower plasma levels of vascular endothelial growth factor (VEGF). Lastly, rats in hyperoxia showed hyperactive and a more explorative behaviour.

**Conclusions:**

Our in vivo infant rat model mimics clinical key features of BPD. To the best of our knowledge, this is the first BPD rat model demonstrating an association between lung structure and function. Moreover, we provide additional evidence that infant rats subjected to hyperoxia develop rarefaction of pulmonary vessels, augmented vascular α-SMA, and adaptive cardiac hypertrophy. Thus, our model provides a clinically relevant tool to further investigate diseases related to O_2_ toxicity and to evaluate novel pharmacological treatment strategies.

**Electronic supplementary material:**

The online version of this article (10.1186/s12967-019-1843-1) contains supplementary material, which is available to authorized users.

## Background

Administration of supplemental oxygen (O_2_) is a cornerstone in the treatment of hypoxaemic critically ill infants and children. In fact, hypoxaemia is a dangerous condition that might end in persistent organ damage and neurological sequelae. While hypoxaemia is feared and well known, noxious effects of excessive O_2_ therapy are generally less recognised [1]. Neonatologists are familiar with clinical consequences of disproportionate O_2_ administration and challenged by bronchopulmonary dysplasia (BPD), a multifactorial chronic lung disease that mainly occurs in premature infants requiring mechanical ventilation and O_2_ therapy [2]. Moreover, BPD is strongly associated with non-favourable long-term cardiovascular and neurological disorders [3–6]. In contrast, paediatric and adult intensivists maintain a rather liberal attitude towards O_2_ therapy despite increasing evidence of harmful systemic effects of hyperoxia in nonhypoxaemic critically ill patients [7]. The current liberal practice is concerning since hyperoxia leads to non-physiologic states favouring oxidative stress [8].

Although experimental BPD models allowed invasive studies that cannot be performed in nonhypoxaemic humans, their translational potential has not been fully explored. Over the last decade, researchers established a variety of animal models to study a broad range of O_2_ concentrations applied over several days. Rats seem to be well suited to model developmental changes encountered in human lungs [9–11]. In particular, infant rat models of hyperoxia closely mimic histological features of the disorganised lung architecture observed in human BPD [12]. Other advantages of using rats include their highly developed social behaviour and relatively large body size, which makes it easier to carry out behavioural experiments, sampling, and surgical manipulations, respectively. In addition, small animal models continue to provide a platform for testing both established and novel treatment strategies for children affected from chronic pulmonary and cardiovascular disease [13, 14].

Despite the above mentioned advantages of hyperoxia-based infant rat models, we argue that their potential has not been maximised, yet. First, besides alveolar simplification, lung fibrosis, and pulmonary vascular remodelling, BPD comes clinically along with impaired respiratory function. However, only few experimental studies have systematically compared structural alterations with sophisticated lung function measurements [15]. Second, traditional histopathologic analyses are prone to less standardised and randomised collection and interpretation of data, particularly when compared to the advantages of image analysis. Third, there is a lack of animal models focusing on extra-pulmonary sequelae of hyperoxia-induced tissue damage.

Hence, the primary aim of our study was to relate alveolar remodelling and fibrosis with respiratory function assessed via image analysis tools and the forced oscillation technique, respectively. We hypothesised an association between these structural alterations and respiratory system mechanics. The second hypothesis was that long-term hyperoxia results in rarefaction of pulmonary vessels, augmented vascular α-SMA, and adaptive cardiac hypertrophy.

## Methods

### Animals

Pregnant Sprague Dawley (SD) dams were purchased from Charles Rivers Laboratories International, Inc. (Sulzfeld, Germany) and delivered on day 14 of pregnancy (E14). Since the average length of the gestation period in rats varies between 21 and 23 days (E21–E23), pregnant dams had at least 1 week of acclimatization, in order to reduce the stress associated with transportation, before the initiation of our experiments. Dams and their pups born in our laboratory facility on day of life (DOL) 0 were housed in individual sealed cages (T1500 IVC) under 12 h light and dark cycle with ad libitum access to water and food, at temperatures of 22–24 °C, and humidity of 30–60%. The litter size varied from 8 to 15 rat pups.

From DOL 1 to 19, dams and their pups were exposed in three separate and consecutive series to room air (0.21), and to a fraction of inspired oxygen (FiO_2_) of 0.6, 0.8, and 1.0. The hyperoxic environment was created by a computer-controlled O_2_ system based on the software IOX (EMKA Technologies, Paris, France). Carbon dioxide (CO_2_) concentrations in the cages were targeted to be below 0.4% and controlled using gas flows of 3–5 Standard Liter Per Minute. Flow rates in the normoxic cages were regulated accordingly via flow regulator Vent2 (EMKA Technologies, Paris, France). O_2_ and CO_2_ concentrations were monitored three times per day using the O_2_ and CO_2_ Datex-Ohmeda sensor (Anandic Medical System, Switzerland). As adult rats do not tolerate chronic exposure of high O_2_ levels, dams were rotated every 24 h between hyperoxic and room air conditions to prevent hyperoxia-associated stress and discomfort. The chambers were daily opened for 10 min to switch the dams, weigh the pups, and clean the cages.

Well-being and social interaction were assessed three times daily and all findings were recorded on a standardised score sheet. Except for daily health checks via observation of hunched posture, piloerection, eye discharge, and reduced social interaction, dams and pups did not experience any physical manipulation until DOL 5, when each pup was tattooed on toes according to the universal rodent numbering system. Tattoos were performed by pricking the skin of a specific finger with a needle dipped in ink (Aramis Laboratory Animal Microtattoo System, Ketchum Manufacturing, Brockville, Canada).

The method of sacrifice of the infant rats at the end of all experiments was maximum blood withdrawal via direct cardiac puncture in a separate room. After euthanasia of all pups, dams were culled in a euthanasia chamber via CO_2_ gas exposure.

### Respiratory system mechanics

On DOL 19, after brief inhalational anaesthesia with isoflurane, infant rats were anaesthetised with an intraperitoneal injection of a solution containing 75 μg/g body weight (BW) of ketamine and 10 μg/g BW of xylazine. After weighing each animal and confirmation of adequate level of anaesthesia via absence of pedal withdrawal reflex, a tracheostomy was performed and a 10 mm polyethylene cannula (ID: 0.86 mm) was inserted. Rats were then placed in supine position on a heating mat and connected to a computer-controlled ventilator (flexiVent^®^, Scireq, Montreal, Canada) using the following settings: fraction of inspired O_2_ (FiO_2_) 0.4 and 1.0 for the normoxic and hyperoxic group, respectively, respiratory rate (RR) 90/min, tidal volume (V_T_) of 8 mL/kg, and positive end-expiratory pressure (PEEP) 5 cm H_2_O. PEEP was regulated by submerging the end of the expiratory tube into a water column. In addition, heart rate, blood pressure, and O_2_ saturation (SpO_2_) were monitored with a small animal pulse oximeter (MouseOx™, STARR Life Sciences Corporation™, Oakmont, PA, USA) by placing a sensor on the proximal part of the thigh.

Next, lung volume history was standardised within 5 min by two lung volume recruitment manoeuvers from 5 cm H_2_O to 40 cm H_2_O with 9 s ramp and 3 s plateau. Then, baseline measurement of respiratory system input impedance (Z_rs_) was performed using the low-frequency forced oscillation technique (FOT) provided by the flexiVent^®^ system. Z_rs_ was obtained with a 6 s oscillation signal of 17 mutually prime frequencies from 0.5 to 19.75 Hz applied to the airway of the infant rat with a PEEP of 5 cm H_2_O to prevent lung derecruitment during Z_rs_ measurements. Thus, oscillations were delivered on top of these PEEP levels. The “constant-phase” model was then fitted to the resulting Z_rs_, allowing the estimation of airway resistance (R_aw_), and the coefficients of tissue damping (G) and elastance (H). Values of R_aw_ were corrected for the resistance of the tracheal cannula. Lung tissue hysteresivity (η) was calculated as the ratio of G and H. After lung function assessments pups underwent terminal blood withdrawal and tissue sampling. Infant rats exposed to FiO_2_ 0.6 did not undergo assessment of respiratory system mechanics due to lack of differences in weight and social behaviour when compared to the normoxic group.

### Sampling and processing of blood

Before disconnecting animals from the ventilator, partial laparotomy and sternotomy were performed and blood was taken via direct cardiac puncture. Blood was collected in plastic tubes containing the anticoagulant EDTA and kept on ice before centrifugation at 3000 rpm for 10 min. Plasma was frozen at − 80 °C for further analysis of endothelin-1 (ET-1), and vascular endothelial growth factor (VEGF) via fluorescence immunoassays. Since VEGF is a marker of impaired vascular development, we did not measure its concentration in the series of experiments using FiO_2_ 0.6 and 0.8 where pulmonary vessels were not analysed histologically.

### Sampling and processing of lung and heart tissues

Lungs of infant rats exposed to FiO_2_ 1.0 were inflated and fixed via tracheal instillation of 4% formalin with a pressure of 10 cm H_2_O. Thirty minutes later, lungs and heart were removed en bloc from the thoracic cavity and stored at − 4 °C in a formalin filled container until histological processing. After fixation for 48 h, lungs and heart were trimmed, dehydrated through graded alcohols and routinely paraffin wax embedded. Consecutive sections (3–5 µm) were prepared, mounted on glass slides and routinely stained with haematoxylin and eosin (H&E), Gomori blue trichrome or subjected to immunohistochemical staining for the detection of smooth muscle and endothelial cells. Briefly, sections were incubated with antibodies against α-SMA (anti-human alpha-smooth muscle actin mouse monoclonal antibody), and von Willebrand Factor (anti-human factor VIII-related antigen (FVIII-Rag) rabbit polyclonal antibody, A0082, Dako-Agilent Technologies, Denmark; 1:100) for 1 h at 37 °C. Afterwards, the slides were incubated for 30 min with a horse radish peroxidase (HRP)-labelled polymer, conjugated to a secondary anti-mouse and anti-rabbit antibody (Dako EnvisionTM System, Dako-Agilent Technologies), respectively. The reaction was visualised using 3,3′-diaminobenzidine (DAB) as chromogen, followed by light counterstain with haematoxylin. The immunohistochemical staining was performed using an Autostainer (Dako Autostainer Universal Staining System Model LV-1, Dako-Agilent Technologies). In addition, heart sections were stained with a fluorescent wheat germ agglutinin to assess cardiomyocyte size. All slides were scanned using a digital slide scanner (NanoZoomer-XR C12000; Hamamatsu, Japan) and histomorphometrical analysis was performed on the digital slides using the Visiopharm Integrator System (VIS, version 4.5.1.324, Visiopharm, Hørsholm, Denmark), unless specified otherwise.

### Lung fibrosis

Myofibroblasts, key effector cells in the development of fibrosis, were identified in lung sections immunostained for anti-α-SMA. α-SMA-positive areas were quantified in each animal using at least 15 fields per section. Results were expressed as fraction of α-SMA-positive areas normalised against the total lung parenchyma excluding the airspace.

### Histomorphometrical study of alveolar remodelling

Alveolar diameters were estimated calculating the mean linear intercept (chord) length (Lm), equal to the mean interalveolar distance, as described previously [16]. In each animal, 10 representative pictures were taken at 40× magnification from the H&E-stained lung sections, avoiding regions with large bronchi. A grid with 11 parallel lines was overlaid onto each image, and the length of each chord was defined by the intercept with the alveolar walls. Mean Lm was calculated by dividing the total length of the line drawn across the lung section by the number of intercepts encountered.

Alveolar counts were determined via Visiopharm software by counting the number of alveoli per field in the H&E-stained sections. In each animal 15 fields per section were analysed at 40× magnification. Briefly, 15 regions of interest (ROIs) with a size of 0.298 mm^2^ were randomly selected from the lung parenchyma in each animal. A threshold classification allowed to distinguish between alveolar lumina and alveolar wall, and to calculate the alveolar count in each ROI.

### Pulmonary arterial medial wall thickness and count of pulmonary vessels

Pulmonary arterial medial wall hypertrophy was assessed at 40× magnification in lung sections immunostained for anti-α-SMA. At least 15 ROIs with a size of 2.605 mm^2^, containing vessels with a diameter of < 100 µm, were randomly selected across the lung parenchyma of all animals, excluding fields containing terminal bronchioles. A threshold classification allowed to distinguish between α-SMA-positive and negative tissue. The results were expressed as α-SMA-positive area per cross sectional vessel. The number of pulmonary vessels was assessed in lung sections immunostained for von Willebrand Factor within the outlined ROIs. A threshold classification allowed to select vessels with a diameter between 30 and 100 µm. Fields containing bronchioles were excluded from the analysis.

### Right ventricular hypertrophy (RVH)

The thickness of the right (RV) and left (LV) ventricular free walls was measured in H&E-stained heart sections using the NDP view software (Hamamatsu Photonics), and the RV/LV ratio was calculated as a marker of RVH. As an additional marker of RVH, the cross-sectional area of cardiomyocytes was assessed at 40× magnification in heart sections stained for anti-WGA (wheat germ agglutinin). A threshold classification allowed the recognition of WGA-stained membrane and empty sarcoplasm in at least 40 representative right ventricular cardiomyocytes with a central 4′,6-diamidino-2-phenylindole (DAPI)-stained nucleus.

### Statistical analysis

Statistical comparisons between the normoxic and hyperoxic group were performed using the t-test. Where satisfaction of normality and equality was not possible the non-parametric Mann–Whitney rank sum test was used. Values are reported as mean ± standard deviation for body weight, and as mean ± standard error of means for all other experimental data. Linear regression was used to examine the association between histological parameters and lung function. The strength of association was expressed as a coefficient of determination, denoted as r^2^. Statistically significant data are additionally expressed as vertical box plots with median, 10th, 25th, 75th, and 90th percentiles. Statistical significance was set at a p-value (p) < 0.05.

## Results

### Social behaviour, well-being, and survival rates

No signs of stress or abnormal behaviour were observed after exposure to FiO_2_ 0.6 and 0.8. In contrast, all infant rats exposed to FiO_2_ 1.0 developed a hyperactive behaviour from DOL 15 onwards. This behaviour was characterised by a pronounced exploration and interaction without signs of self-inflicted injuries or aggression towards littermates. There were no deaths.

### Postnatal growth

**Fig. 1:**
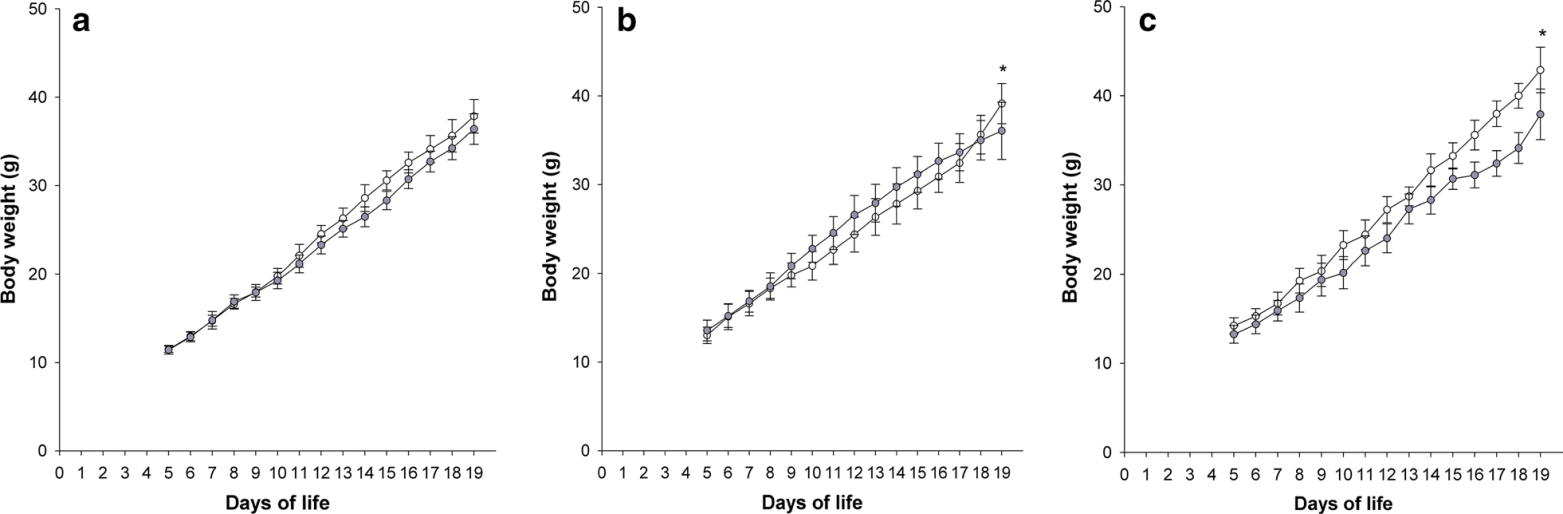
Body weight gain from DOL 5 to 19. Curves with white and grey circles indicate the normoxic and FiO_2_ 0.6 (**a**), FiO_2_ 0.8 (**b**), and FiO_2_ 1.0 (**c**) groups, respectively. Asterisk displays a significant difference on DOL 19 between study groups, p < 0.05

Exposure to FiO_2_ 0.6 did not affect weight gain in the hyperoxic group compared to normoxic controls (p = 0.645). In contrast, application of FiO_2_ 0.8 resulted in a significant weight difference on DOL 19 with normoxic (n = 15) and hyperoxic (n = 14) animals weighing 39.1 ± 8 g and 36.1 ± 8 g, respectively (p = 0.041). A higher difference in weight was found in the FiO_2_ 1.0 series with normoxic (n = 8) and hyperoxic (n = 8) infant rats weighing 42.9 ± 1.9 g and 38.0 ± 3.1 g, respectively (p < 0.001) (Fig. 1).

### Respiratory system mechanics

**Fig. 2:**
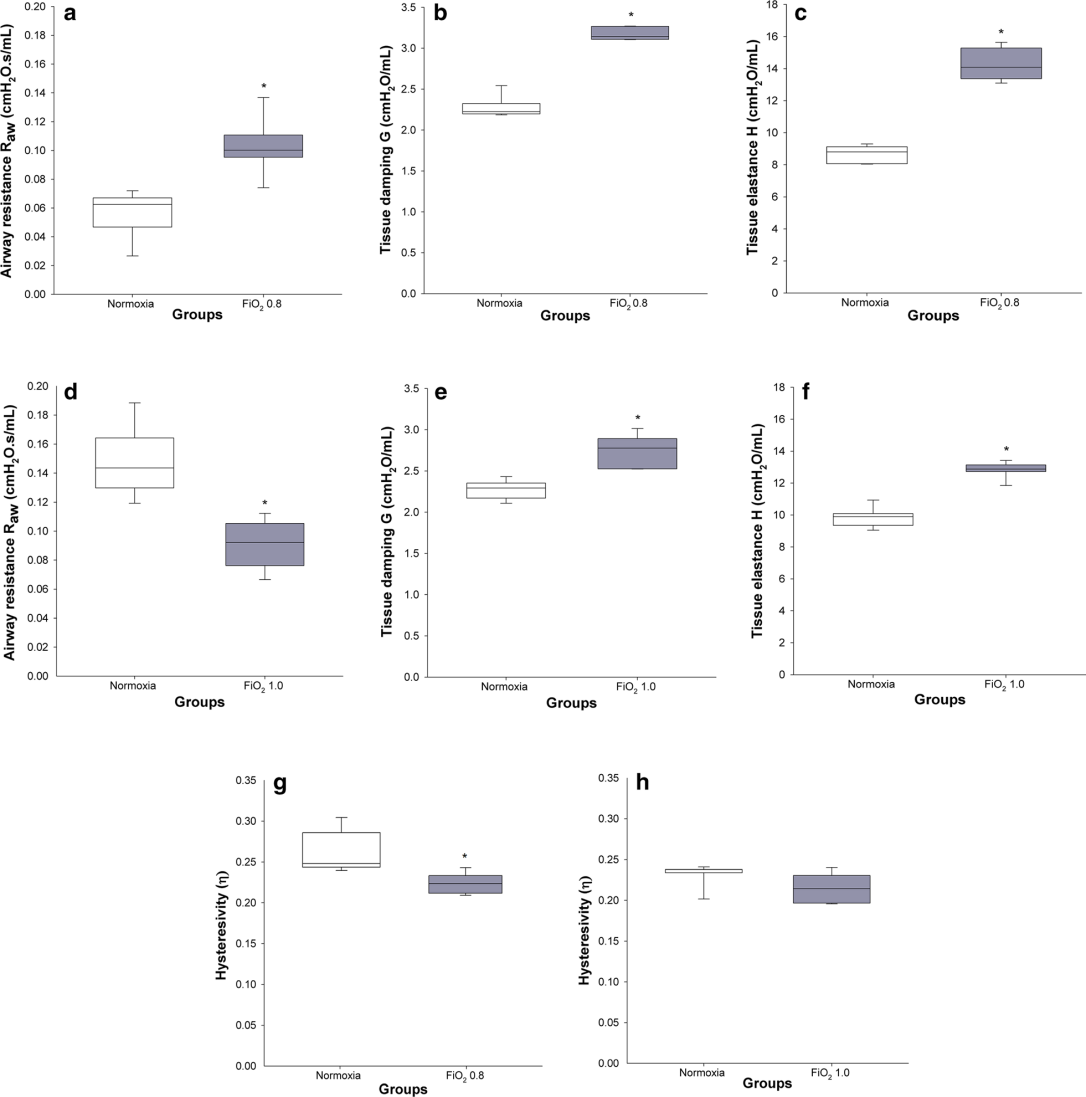
Respiratory system mechanics on DOL 19. Normoxic and hyperoxic study groups are illustrated with white and grey box plots, respectively. **a**–**c**, **g** R_aw_, G, H, and η in normoxia (n = 6) and hyperoxia (FiO_2_ 0.8) (n = 6). **d**–**f**, **h** R_aw_, G, H, and η in normoxia (n = 7) and hyperoxia (FiO_2_ 1.0) (n = 6). R_aw_: airway resistance; G: coefficient of tissue damping; H: coefficient of tissue elastance; η: lung tissue hysteresivity. Data are expressed as vertical box plots with median, 10th, 25th, 75th, and 90th percentiles. Asterisk displays a significant difference between study groups, p < 0.05

A significantly higher airway resistance R_aw_ was found in rats exposed to FiO_2_ 0.8 when compared to the control group (0.10 ± 0.01 cm H_2_O s/mL versus 0.06 ± 0.01 cm H_2_O s/mL) (p = 0.002). In contrast, exposure to FiO_2_ 1.0 resulted in significantly lower R_aw_ (0.09 ± 0.01 cm H_2_O s/mL) in comparison with the normoxic group (0.15 ± 0.01 cm H_2_O s/mL) (p < 0.001) (Fig. 2).

The coefficient of tissue damping G significantly increased by 38% and 21% in FiO_2_ 0.8 and 1.0, respectively, when compared to the respective normoxic groups (in both cases p < 0.001) (Fig. 2). A similar pattern was found for the coefficient of tissue elastance H, where FiO_2_ 0.8 and 1.0 resulted in 64% and 30% higher H, respectively, when compared to the control groups (in both cases p < 0.001) (Fig. 2). Lung tissue hysteresivity η was 18% and 7% lower after exposure to FiO_2_ 0.8 (p = 0.021) and 1.0 (p = 0.132), respectively, in comparison with the groups in normoxia.

### Histology

#### Lung histology and morphometric analysis

**Fig. 3:**
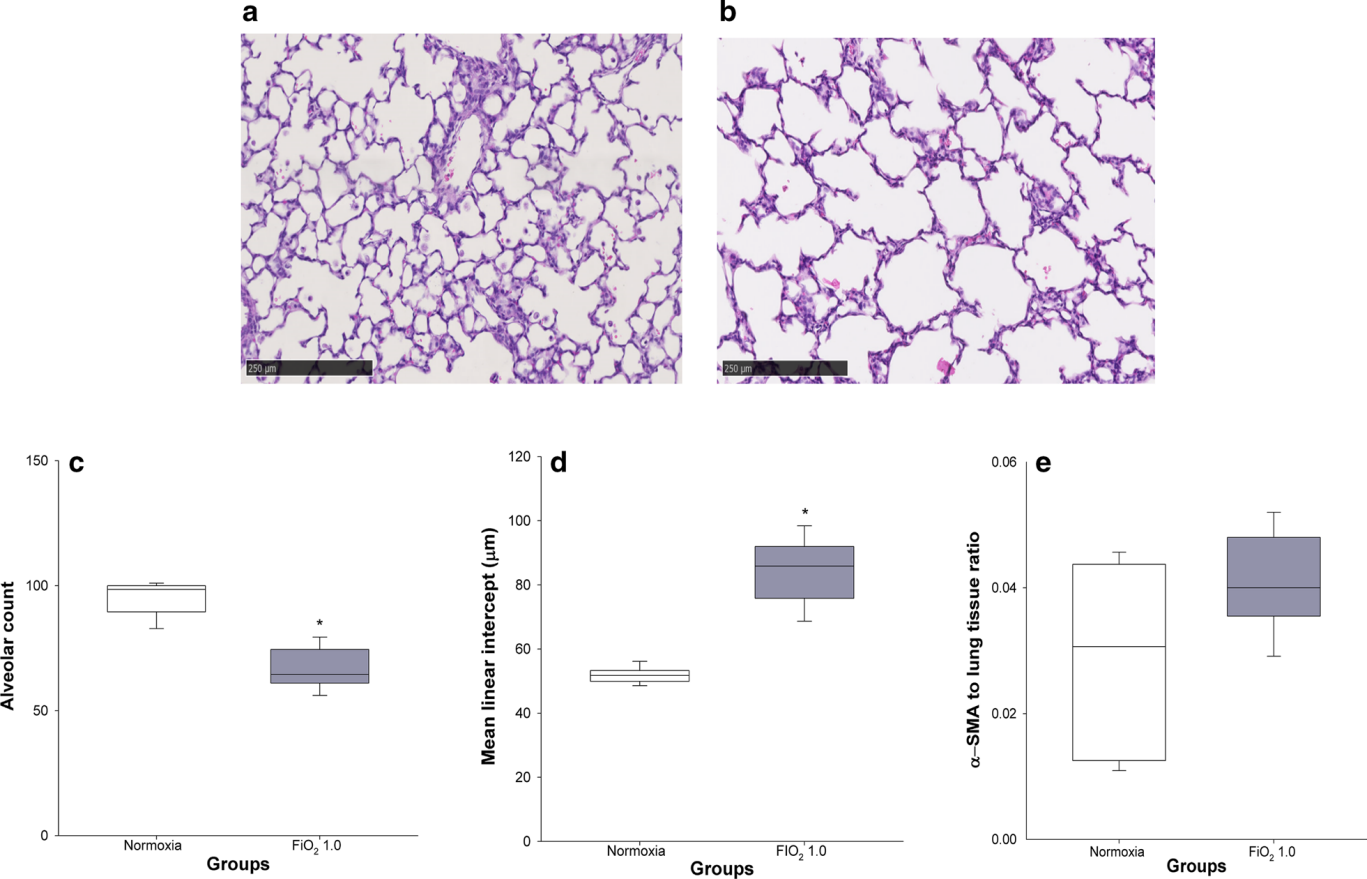
Lung histology and morphometric analysis. Normoxic and hyperoxic study groups are illustrated with white and grey box plots, respectively. **a**, **b** Representative microscopic photographs of lung sections in normoxia (**a**) and hyperoxia (**b**). Magnification ×10. **c**–**e** Alveolar count, mean linear intercept, and ratio of α-SMA-positive lung tissue area/total parenchymal tissue area in normoxia (n = 8) and hyperoxia (n = 8). Data are expressed as vertical box plots with median, 10th, 25th, 75th, and 90th percentiles. Asterisk displays a significant change between study groups, p < 0.05

In comparison with control animals held at room air (Fig. 3a), lung histology of rats exposed to FiO_2_ 1.0 was characterised by alveolar simplification, with fewer and larger alveoli (Fig. 3b). In particular, the hyperoxic group showed a significantly lower alveolar count per field (67 ± 3 vs 95 ± 3, p < 0.001) (Fig. 3c) and higher mean alveolar intercept (84 ± 4 vs 52 ± 1 µm, p < 0.001) (Fig. 3d). On the contrary, no significant differences in lung α-SMA content were found between normoxia (0.029 ± 0.006) and hyperoxia (0.041 ± 0.003) (p = 0.234) (Fig. 3e).

### Histology of pulmonary vessels and heart

**Fig. 4:**
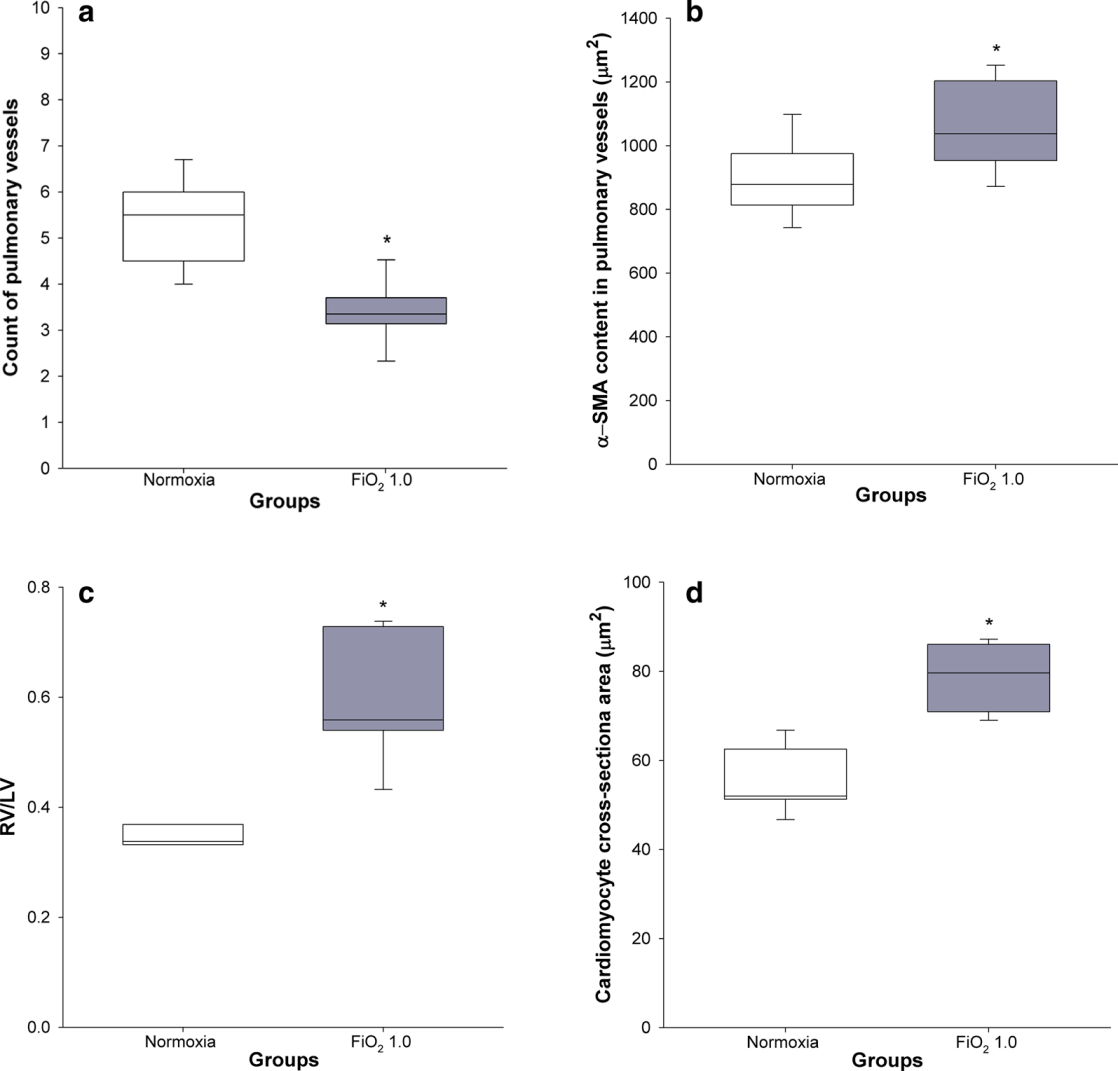
Histomorphometry of pulmonary vessels and heart. Normoxic and hyperoxic study groups are illustrated with white and grey box plots, respectively. **a**–**d** Count of pulmonary vessels (n = 8 + 8), α-SMA content in the medial wall of the pulmonary arterioles (n = 7 + 8), right-to-left ventricle wall thickness ratio (n = 3 + 6), cardiomyocyte cross-sectional area (n = 7 + 8) in normoxia and hyperoxia, respectively. Data are expressed as vertical box plots with median, 10th, 25th, 75th, and 90th percentiles. Asterisk displays a significant difference between study groups, p < 0.05

Hyperoxia led to a significantly lower count of pulmonary arterioles per field (3.5 ± 0.2) in comparison with the normoxic group (5.9 ± 0.3) (p < 0.001) (Fig. 4a). In addition, we observed a significant difference in the hyperoxic group, when compared to normoxic controls, with regard to α-SMA content in the medial wall of pulmonary vessels (1099 ± 46 versus 904 ± 50 µm^2^, p = 0.013) (Fig. 4b), RV/LV ratio (0.59 ± 0.05 versus 0.35 ± 0.01, p = 0.001) (Fig. 4c), and cross-sectional area of the right ventricular cardiomyocytes (78.9 ± 3.0 µm^2^ vs 55.3 ± 2.9 µm^2^, p < 0.001) (Fig. 4d).

### Structure–function relation

**Fig. 5:**
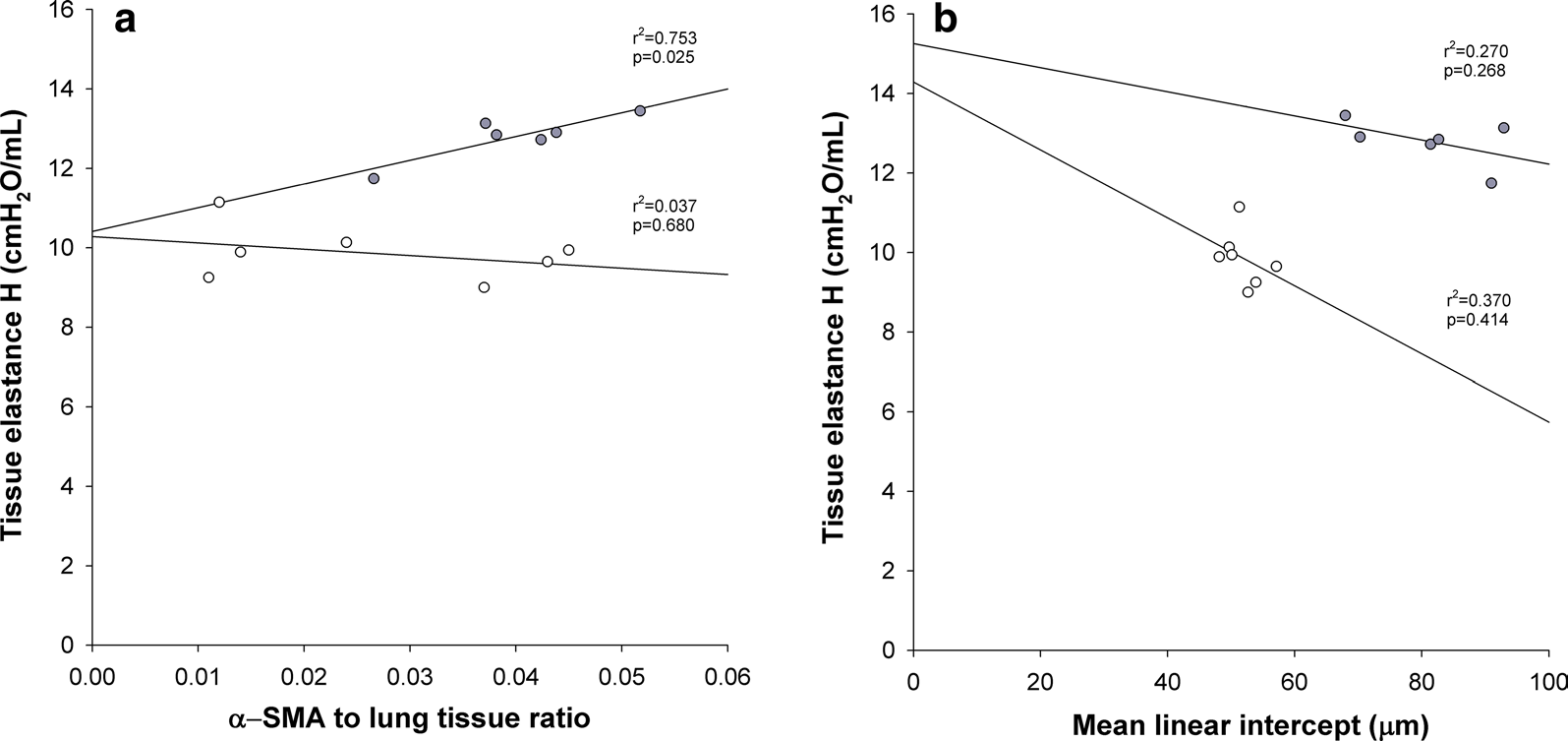
Scatter plot of α-SMA to lung tissue ratio (**a**) and mean linear intercept (**b**) against lung tissue elastance (H). White and grey circles indicate the normoxic (n = 7) and FiO_2_ 1.0 (n = 6) groups, respectively; r^2^ = coefficient of determination

A significant association between the content of α-SMA in lung parenchyma and tissue elastance H was observed in the hyperoxic study group (r^2^ = 0.753, p = 0.025) (Fig. 5a). No association was found between mean linear intercept (Lm) and H, in both normoxia (p = 0.414) and hyperoxia (p = 0.268) (Fig. 5b).

### Biomarkers

Biomarkers were measured at a single time point of DOL 19.

### VEGF and ET-1 plasma concentrations

**Fig. 6:**
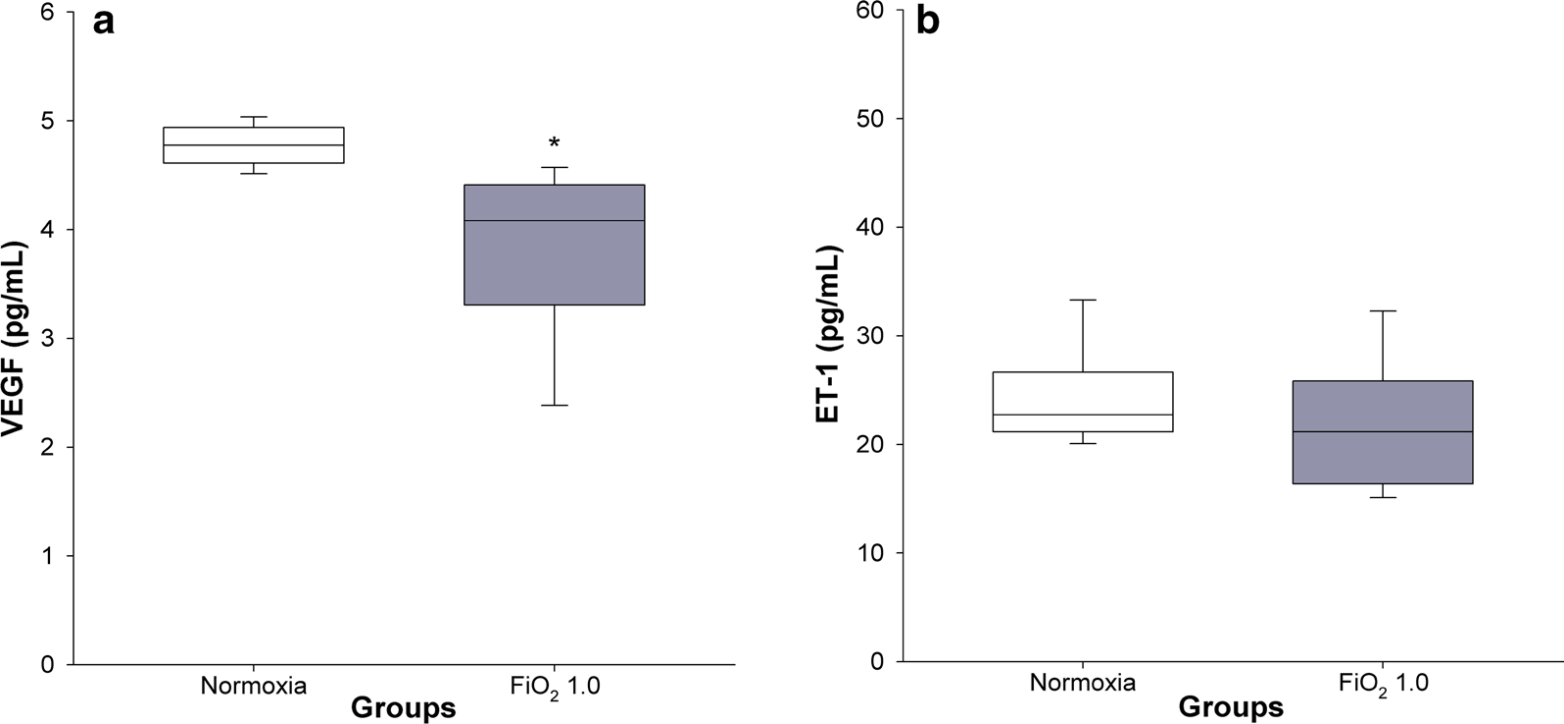
VEGF and ET-1 concentrations in plasma. Normoxic and hyperoxic study groups are illustrated with white and grey box plots, respectively. **a** VEGF concentration in normoxia (n = 8) and hyperoxia (FiO_2_ 1.0) (n = 8). **b** ET-1 concentration in normoxia (n = 8) and hyperoxia (n = 7). Data are expressed as vertical box plots with median, 10th, 25th, 75th, and 90th percentiles. Asterisk displays a significant change between study groups, p < 0.05

Hyperoxia resulted in significantly lower plasma levels of VEGF (3.7 ± 0.3 pg/mL) in comparison with the normoxic group (4.8 ± 0.1 pg/mL) (p = 0.006), (Fig. 6a). No significant differences in ET-1 concentration were found between normoxia (24.5 ± 1.8 pg/mL) and hyperoxia (21.4 ± 2.6 pg/mL) (p = 0.417) (Fig. 6b).

## Discussion

This study provides an infant rat model of hyperoxia-induced tissue damage mimicking clinical key features of BPD. In line with our first hypothesis and as far as we know, this is the first BPD rat model demonstrating an association between lung structure and function using accurate diagnostic tools such as image analysis and FOT, respectively. Moreover, according to our second hypothesis, we reproduced pronounced rarefaction of pulmonary vessels, augmented vascular α-SMA, and adaptive cardiac hypertrophy.

Rats have a high translational value because they are born at a lung developmental stage (saccular stage) equivalent to that of an extreme premature infant and reliably reproduce structural changes closely mimicking human BPD [11, 12]. However, in our view the potential of BPD models has not been maximised, yet. In fact, although O_2_ is the most commonly applied injurious stimulus for inducing pulmonary hallmark features of BPD [9], the use of different O_2_ concentrations and time of exposure to reproduce key features of this disease has generated data difficult to compare [15].

Hence, our group first designed an O_2_-response study consisting of three separate and consecutive series where infant rats were exposed to FiO_2_ of 0.6, 0.8, and 1.0. FiO_2_ 0.6 was insufficient to induce clinically relevant morbidity. On the contrary, the treatment with FiO_2_ 1.0 resulted in earlier and more pronounced lower body weights than FiO_2_ 0.8 as well as abnormal behaviour, in line with behavioural mice studies [17], when compared to normoxic controls.

The properties of the respiratory system mechanics were characterised via FOT. The high R_aw_ after exposure to FiO_2_ 0.8 (Fig. 2a) was in line with previous studies where hyperoxia led to remodelling of conducting airways and smooth muscles, and to increased airway hyperresponsiveness in infant and adult rodents [9, 18, 19]. Surprisingly, exposure to FiO_2_ 1.0 resulted in lower R_aw_ compared to normoxic controls (Fig. 2d). Since we did not assess structural changes of the airways, we can only speculate whether stress-related glucocorticoid and catecholamine release [20], though not perceivable by the experimenter, resulted in bronchodilation outweighing remodelling of the airways. On the other hand, a study performed in a BPD rabbit model showed no significant differences in R_aw_ between normoxic and hyperoxic animals questioning the airway remodelling hypothesis [21].

In agreement with comparable experimental studies [21, 22], tissue damping G and tissue elastance H showed significantly higher values after exposure to hyperoxia (Fig. 2). Since G and H exhibit inverse dependencies on body weight [23], we supposed that higher G and H after application of FiO_2_ 0.8 result from combined effects of hyperoxia and lower body weights. While G is closely related to tissue resistance and regional heterogeneity [24], common findings in BPD [2], H reflects tissue stiffness and reduced compliance [25]. Although the levels of α-SMA were not significantly increased in hyperoxia, we observed a ~ 40% higher concentration of this biomarker in hyperoxic animals (Fig. 3e). The accumulation of α-SMA in lung tissue reflects the differentiation of lung fibroblasts into myofibroblasts [26], which play a major role in the pathogenesis of pulmonary fibrosis [27].

Lung tissue hysteresivity (η) is defined as the energy dissipated (G) relative to the elastic energy stored in the lung (H) [28]. In case of moderate to severe heterogeneity, G increases proportionally more than H [29–31]. In contrast, lung derecruitment comes along with a proportionate increase of G and H [31, 32]. Hence, η will rise or remain unchanged. In our study, a more pronounced increase of H in respect to G led to a decrease of η both after exposure to FiO_2_ 0.8 and FiO_2_ 1.0 (Fig. 2). This finding was unexpected. In theory, a fall in η can occur when H rises proportionally more than G or when H decreases to a lesser extent compared to G. The second scenario can be excluded, since we did neither observe lower values of H nor G in hyperoxia. Hence, in our view, the behaviour of η predominantly reflected progressive lung volume derecruitment accompanied by a consecutive lung heterogeneity questioning the association between stable η and substantial derecruitment [31, 32].

Studies exploring the relationship between alterations in lung structure and functional impairment of respiratory mechanics in animal models of BPD are lacking [15]. Therefore, we related our histopathologic findings to changes in respiratory system mechanics. In contrast to experimental studies in emphysema rodent models [33, 34], our linear regression analysis did not show an association between Lm, the most commonly used index of airspace enlargement, and H (Fig. 5b). In our view, this finding is not surprising since BPD, as opposed to emphysema, cannot be considered solely an obstructive lung disease. In fact and consistent with the behaviour of η, a linear relationship was detected between lung parenchymal α-SMA content and H (Fig. 5a), mirroring both loss of lung volume and a significant restrictive component in BPD.

In accordance with former rat BPD studies [35], we also found a significant rarefaction of pulmonary vessels (Fig. 4a) and higher concentration of α-SMA in smooth muscle cells of the pulmonary arteriolar wall (Fig. 4b). Significantly higher levels of the right-to-left ventricle diameter ratio (Fig. 4c) and cardiomyocyte cross-sectional area (Fig. 4d), both markers of RVH, can be interpreted as a consequence of pulmonary vascular structural alterations leading to increased afterload, a common complication of BPD [6].

In agreement with comparable human BPD and animal studies [36, 37], we found that exposure to FiO_2_ 1.0 led to a significantly lower VEGF concentration in plasma (Fig. 6a). Measurement of the second biomarker ET-1, involved in the pathogenesis of pulmonary vascular disease [38], did not differ between hyperoxic and control groups (Fig. 6b). Baumann et al. [39] found a significant difference in a precursor of ET-1 in BPD-affected children until 28 days of age only. However, this difference vanished at 36 weeks postmenstrual age. From a translational point of view, 19 days old infant rats can be compared to preschool children. Hence, it is not surprising that at the time where we measured ET-1 no significant differences between study groups were observed.

This study is subject to limitations. First, although we inferred lung volume derecruitment from H values, we did not perform direct lung volume measurements. Second, we assumed that glucocorticoids and catecholamines were released by the neuroendocrine system to contrast stress. However, we neither assessed the concentration of stress hormones nor the airway calibre. Third, we did not perform standardised observational tests. In fact, our well-being score sheet was not designed to adequately capture hyperactive and explorative behaviour. Last, although O_2_ represents the most commonly applied injurious trigger for inducing key features of BPD, it has to be taken into account that the etiology of BPD is multifactorial. Therefore, to truly mirror the multifactorial clinical and genetic conditions contributing to human BPD, an ideal animal model would aim at combining multiple factors [9]. This remains a substantial limitation, which can only partially be overcome by optimizing study methods.

## Conclusions

To our knowledge, this is the first BPD rat model demonstrating an association between pulmonary structural and functional changes using accurate diagnostic tools such as image analysis and FOT, respectively. Moreover, we provide additional evidence that infant rats subjected to hyperoxia develop rarefaction of pulmonary vessels, augmented vascular α-SMA, and adaptive cardiac hypertrophy. Hence, the present in vivo study provides a clinically relevant model to further investigate pathogenesis of diseases related to O_2_ toxicity and to evaluate novel pharmacological treatment strategies (Additional file 1: Figure S1).

## Additional file

**Additional file 1: Figure S1.** ET-1 concentration in plasma. Normoxic and hyperoxic study groups are illustrated with white and grey box plots, respectively. **A**: ET-1 concentration in the normoxic (n = 13) and FiO_2_ 0.6 groups (n = 14). **B**: ET-1 concentration in normoxia (n = 15) and hyperoxia (FiO_2_ 0.8) (n = 14). Data are expressed as vertical box plots with median, 10th, 25th, 75th, and 90th percentiles.

## Abbreviations

α-SMA: alpha-smooth muscle actin
BPD: bronchopulmonary dysplasia
BW: body weight
DOL: day of life
ET-1: endothelin-1
Eta (η): tissue hysteresivity
FiO_2_: fraction of inspired oxygen
FOT: forced oscillation technique
G: tissue damping
H: tissue elastance
HIF-2: hypoxia-inducible factor-2
Lm: mean linear intercept length
LV: left ventricle
n: number of animals
PEEP: positive end-expiratory pressure
R_aw_: airway resistance
ROI: region of interest
RR: respiratory rate
RV: right ventricle
RVH: right ventricular hypertrophy
SD: Sprague–Dawley
VEGF: vascular endothelial growth factor
V_T_: tidal volume
Z_rs_: respiratory system impedance
