# Supplementary material for: Hyperoxia-induced lung structure–function relation, vessel rarefaction, and cardiac hypertrophy in an infant rat model
Source: J Transl Med. 2019 Mar 18;17:91. doi: 10.1186/s12967-019-1843-1 (PMC6423834; doi:10.1186/s12967-019-1843-1)
Supplement: Supplementary file 1 — Additional file 1: Figure S1. ET-1 concentration in plasma. Normoxic and hyperoxic study groups are illustrated with white and grey box plots, respectively. A: ET-1 concentration in the normoxic (n = 13) and FiO2 0.6 groups (n = 14). B: ET-1 concentration in normoxia (n = 15) and hyperoxia (FiO2 0.8) (n = 14). Data are expressed as vertical box plots with median, 10th, 25th, 75th, and 90th percentiles. [file 12967_2019_1843_MOESM1_ESM.docx]

**A B**


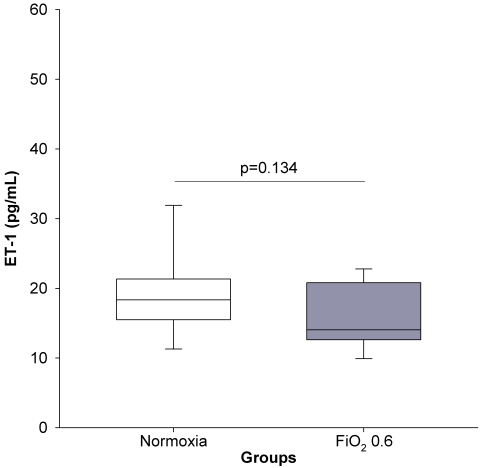

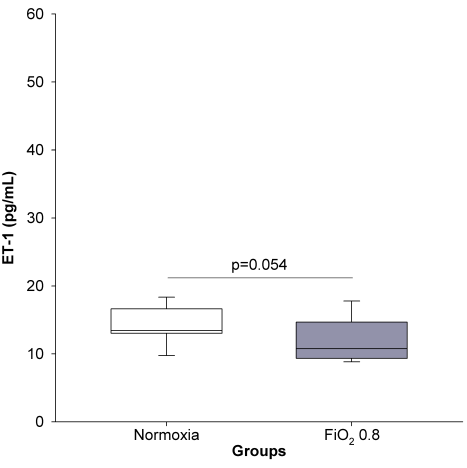


**Figure S1.** ET-1 concentration in plasma. Normoxic and hyperoxic study groups are illustrated with white and grey box plots, respectively. **A:** ET-1 concentration in the normoxic (n=13) and FiO_2_ 0.6 groups (n=14). **B:** ET-1 concentration in normoxia (n=15) and hyperoxia (FiO_2_ 0.8) (n=14). Data are expressed as vertical box plots with median, 10th, 25th, 75th, and 90th percentiles.
